# 

**Published:** 1906-01-06

**Authors:** 


### NOTICE TO CORRESPONDENTS.

All MSS., Letters, Books for Review, and other matters intended for the Editor should be addressed THE EDITOR, "THE HOSPITAL," THE HOSPITAL BUILDINGS, 28 & 29 SOUTHAMPTON STREET, STRAND, W.C.

The Editor cannot undertake to return rejected MS., even when accompanied by stamped directed envelope.

**Notice to Advertisers and Subscribers.**

All Advertisements, Orders for Copies of THE HOSPITAL, and Business Communications should be addressed to **THE MANAGER (Not to the Editor),** THE HOSPITAL, 28 & 29 Southampton Street, Strand London, W.C.

The Cost of Subscription, post free, is as follows:—Per week, 3½d.; per quarter, 3s. 3d.; per half-year, 6s. 6d.; per annum, 13s. Foreign and Colonial:—Per annum, 19s. 6d., payable, in all cases, in advance.

**NOTICE.—Vol. XXXVIII. of "The Hospital" April to September 1905), in handsome cloth binding, will shortly be on sale at the office of this Journal, price 10s. 6d. Cases for binding the Numbers contained in this Volume 2s. Index to Vol. XXXVIII., 3½d., post free.**

**The Monthly Part for January is now ready. Price 1s., by post, 1s. 3d.**

### BOOKS RECEIVED.

Hodder and Stoughton.

Medical Epitome Series:—

"Anatomy." By H. E. Hale, M.D.
"Physiology." By T. C. Guenther, M.D., and A. E. Guenther, B.S.
"Inorganic Chemistry and Physics." By A. McGlannan, M.D.
"Medical Diagnosis." By A. W. Hollis, M.D.
"Clinical Diagnosis and Uranalysis." By J. R. Arneill, M.D.
"Nervous and Mental Diseases." By J. D. Nagel, M.D.
"Histology." By J. R. Wathen, M.D.
"Pathology." By J. Stenhouse, M.D.
"Microscopy and Bacteriology." By P. E. Archinard, M.D.
"Surgery." By M. D. Magee, M.D., and W. Johnson, M.D.
"Eye and Ear." By A. N. Alling, M.D., and O. A. Griffin, M.D.
"Genito-Urinary and Venereal Diseases." By L. E. Schmidt, M.D.
"Diseases of the Skin." By A. Schalek, M.D.
"Obstetrics." By W. P. Manton, M.D.
"Pediatrics." By H. E. Tuley, M.D.
"Toxicology." By E. W. Dwight, M.D.

Dale, Reynolds and Co., Ltd.

"On Active Service in South Africa." By W. S. Inder.

### MEDICAL APPOINTMENTS AND VACANCIES.

### VACANCIES.

BIRMINGHAM, UNIVERSITY OF.—Demonstratorship in Anatomy.

BRISTOL ROYAL INFIRMARY.—Pathologist, Bacteriologist, and Director of Clinical Laboratories.

BRISTOL, UNIVERSITY COLLEGE, FACULTY OF MEDICINE.—Professor of Pathology.

DERBYSHIRE ROYAL INFIRMARY.—Two House Surgeons, one House Physician, and one Assistant House Surgeon.

HOSPITAL FOR CONSUMPTION AND DISEASES OF THE CHEST, Brompton.—Resident House Physicians.

LONDON FEVER HOSPITAL, Liverpool Road, N.—Resident Medical Officer.

MIDDLESBROUGH, NORTH RIDING INFIRMARY.—Assistant House Surgeon.

NATIONAL HOSPITAL FOR THE PARALYSED AND EPILEPTIC, Queen Square, Bloomsbury, W.C.—Resident Medical Officer.

ROYAL FREE HOSPITAL, Gray's Inn Road, W.C.—Medical Registrar; also a Surgical Registrar (females).

ST. BARTHOLOMEW'S HOSPITAL.—Assistant Physician.

SAMARITAN FREE HOSPITAL FOR WOMEN, Marylebone Road, N.W.—Clinical Assistants.

SEAMEN'S HOSPITAL SOCIETY, Greenwich.—Two Assistant Physicians and one Assistant Physician or Surgeon for Diseases of the Throat, Nose, and Ears; also Pathologist.

SOUTHPORT INFIRMARY.—Resident Junior House and Visiting Surgeon.

UNIVERSITY OF LONDON.—Professorship of Protozoology.

VICTORIA HOSPITAL FOR CHILDREN, Tite Street, Chelsea, S.W.—House Surgeon for six months.

WOLVERHAMPTON AND STAFFORDSHIRE GENERAL HOSPITAL.—Assistant House Physician for six months.

### THE NURSE'S BOOKSHELF

**HOW TO BECOME A NURSE:**
**How and Where to Train.**

Edited by Sir Henry Burdett, K.C.B.

A complete guide to the Nursing Profession.

2/- net, 2/4 post free.

**The Duties of a Nurse Explained.**
**NURSING HINTS TO PROBATIONERS ON PRACTICAL WORK.**

By M. Annesley Voysey.

2/- net, 2/3 post free.

The *Lancet* says: "This is a very good practical book. . . . It contains the whole duty of Nurses given in plain language."

**PRACTICAL GUIDE TO SURGICAL BANDAGING AND DRESSINGS.**

By Wm. Johnson Smith, F.R.C.S.

Suitable size for Apron Pocket. 2/- post free.

**THE NURSE'S PRONOUNCING DICTIONARY OF MEDICAL TERMS AND NURSING TREATMENT.**

Compiled by Honnor Morten.
New Edition edited by Mary I. Burdett.
Suitable size for apron pocket, 2/- post free.

**OPHTHALMIC NURSING.**

By Sydney Stephenson, M.B., F.R.C.S.

New and Revised Edition. 3/6 net, 3/10 post free.

**THE MODERN NURSING OF CONSUMPTION.**

By Dr. Jane Walker.
1/- net, 1/2 post free.

**SURGICAL WARD-WORK AND NURSING.**

By Alex. Miles, M.D.
Illustrated. Cloth, 3/6 net, 3/10 post free.

**HOSPITAL SISTERS AND THEIR DUTIES.**

By Eva C. E. Lückes, Matron, London Hospital.
Third Edition, 2/6 post free.

**SIMPLE SANITATION:**
**The Practical Application of the Laws of Health to Small Dwellings.**

By M. Loane. Cloth 1/- net, 1/2 post free.

**THE EXAMINATION OF THE URINE.**

By J. K. Watson, M.D.
Cloth, 1/- net, 1/1 post free.

### THE BURDETT SERIES.

Bound in Cloth Boards, 1/- each, post free.

**PRACTICAL HINTS ON DISTRICT NURSING.**
By Amy Hughes, General Superintendent, Queen Victoria's Jubilee Nurses.

**HOSPITAL AND PRIVATE NURSING.**
By S. S. Orme.

**THE MIDWIVES' POCKET BOOK.**
By Honnor Morten.

**CARE OF CONSUMPTIVES.**
By Dr. W. H. Daw.

**FEVERS AND INFECTIOUS DISEASES.**
By Dr. William Harding.

**HOME NURSING OF SICK CHILDREN.**
By Dr. J. D. E. Mortimer.

**MENTAL NURSING.**
By Dr. William Harding, Superintendent Berry Wood Asylum, Northampton.

**SICK NURSING AT HOME.**
By G. Moberly.

**GYNÆCOLOGICAL NURSING.**
By Dr. Hawkins Ambler.

**SYLLABUS OF LECTURES TO NURSES.**
By Dr. Andrew Davidson.

*Complete Catalogue of Nursing Manuals, Case Books, Charts, &c., post free on application.*

### THE SCIENTIFIC PRESS, LTD.,
**28 & 29 Southampton Street, Strand,**
**LONDON.**

